# Cardiovascular patches applied in congenital cardiac surgery: Current materials and prospects

**DOI:** 10.1002/btm2.10706

**Published:** 2024-09-30

**Authors:** Mingze Sun, Vincent Reed LaSala, Caroline Giuglaris, David Blitzer, Sophia Jackman, Senay Ustunel, Kavya Rajesh, David Kalfa

**Affiliations:** ^1^ Department of Surgery Columbia University New York New York USA; ^2^ UMR 168 Laboratoire Physique des Cellules et Cancer Institut Curie, PSL Research University, Sorbonne Université, CNRS Paris France; ^3^ Division of Cardiac, Thoracic and Vascular Surgery, Section of Pediatric and Congenital Cardiac Surgery New‐York Presbyterian—Morgan Stanley Children's Hospital, Columbia University Irving Medical Center New York New York USA

**Keywords:** biological, cardiovascular patches, congenital cardiac surgery, pediatric surgery, polymeric, tissue engineering

## Abstract

Congenital Heart Defects (CHDs) are the most common congenital anomalies, affecting between 4 and 75 per 1000 live births. Cardiovascular patches (CVPs) are frequently used as part of the surgical armamentarium to reconstruct cardiovascular structures to correct CHDs in pediatric patients. This review aims to evaluate the history of cardiovascular patches, currently available options, clinical applications, and important features of these patches. Performance and outcomes of different patch materials are assessed to provide reference points for clinicians. The target audience includes clinicians seeking data on clinical performance as they make choices between different patch products, as well as scientists and engineers working to develop patches or synthesize new patch materials.

**Translational Impact Statement**

Congenital Heart Defects (CHDs) are the most common congenital anomalies, affecting between 4 and 75 per 1000 live births. This comprehensive review on cardiovascular patches (CVPs) for treating Congenital Heart Defects (CHDs) in children presents an essential guide for clinicians, engineers, and researchers. It explores the evolution, efficacy, performance, properties and selection of CVPs, emphasizing adaptation to pediatric needs for improved surgical outcomes and addressing the critical gap in current congenital cardiac care.

**Abbreviations**
ACEV: aortic cusp extension valvuloplasty
AI: aortic insufficiency
AS: aortic stenosis
ASD: atrial septal defect
AV: aotic valve
AVSD: atrioventricular septal defect
CHD: congenital heart defect
CoA: coarctation of the aorta
CVP: cardiovascular patch
DILV: double inlet left ventricle
DORV: double outlet right ventricle
d‐TGA: dextro‐transposition of the great arteries
ePTFE: expanded polytetrafluoroethylene
GA: glutaraldehyde
GRF: gelatine‐resorcine‐formol
HLA: human leukocyte antigen
HLHS: hypoplastic left heart syndrome
IVC: inferior vena cava
LVOTO: left ventricular outflow tract obstruction
MV: mitral valve
P3HB: poly(3‐hydroxybutyrate)
PA: pulmonary artery
PCL: poly(e‐caprolactone)
PGA: poly(glycolic acid)
PLCL: poly(l‐lactide‐co‐ε‐caprolactone)
PLGA: poly(lactic‐co‐glycolic acid)
PTFE: polytetrafluoroethylene
PU: polyurethane
PV: pulmonary valve
RV: right ventricle
RVOT: right ventricular outflow tract
SIS: small intestinal submucosa
SIS‐ECM: small intestinal submucosa extracellular matrix
SVC: superior vena cava
TE: tissue engineered
TOF: tetralogy of fallot
TV: tricuspid valve
VSD: ventricular septal defect

## 1 INTRODUCTION

Congenital heart defects (CHDs) comprise problems with the structure of the heart present at birth and are the most common type of birth defect. In the United States nearly 1% of infants, 40,000 per year, are born with CHDs.^1^, ^2^, ^3^ Infants with critical CHDs generally need surgery or other procedures early in life, and many of these procedures require the use of cardiovascular patches (CVPs) made of foreign materials to reconstruct cardiovascular structures.

**FIGURE 1:**
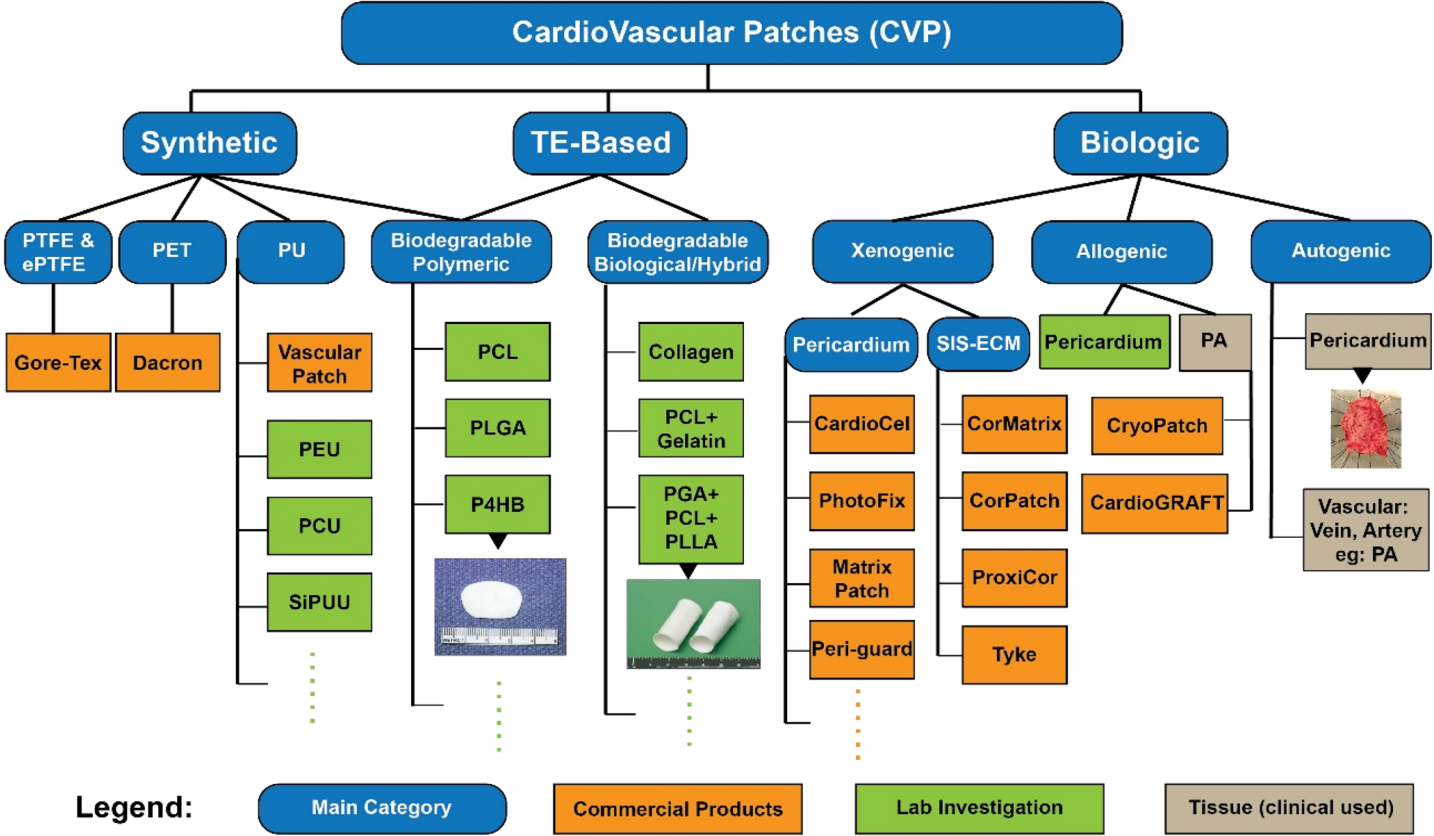
Scheme to illustrate different types of cardiovascular patches. Image of P4HB patch was reprinted from Reference [29], copyright (2000), with permission from Elsevier. Image of “PGA + PCL + PLLA” patch was reprinted from Reference [30], copyright (2005), with permission from Elsevier. Image of autologous pericardium patch was reprinted from Reference [45] with unrestricted use.

Beginning in the late 1950s, various materials such as polyester, polytetrafluoroethylene (PTFE), bovine pericardium, and autologous pericardium have been used in preclinical and clinical trials, summarized in Figure 1, with mixed short and long‐term results. Patches made of synthetic polymers offer the advantage of being readily available (ready to use and easily stored), but with an increased risk of thrombogenicity, infection, bleeding, and aneurysm. In 1953, Michael DeBakey performed the first successful Dacron graft replacement of the thoracic aorta and started an era of utilizing polymer grafts in the treatment of the diseased cardiovascular system.^4^ In 1976, Florian et al.^5^ reported the use of various types of PTFE for small vessel replacement in dogs and found that a high porosity expanded PTFE (ePTFE) yielded the best patency in canine arteries. In the 1990s, Nistal et al. implanted an ePTFE valve prosthesis in the tricuspid position of sheep with survival up to 34 weeks, while Fietsam et al. successfully used an ePTFE patch for angioplasty after canine carotid endarterectomy.^6^, ^7^ Since then, ePTFE has been successfully applied for several clinical applications, such as repair of the aortico‐left ventricular tunnel, carotid patch angioplasty, and right ventricular outflow tract (RVOT) reconstruction.^8^, ^9^, ^10^

Biological patches were developed in parallel. These patches serve as an alternative option, providing greater resistance to infection and thrombosis with minimal suture line bleeding. Two types of biological patches, pericardium and small intestinal submucosa (SIS), have been commercialized and widely used. In the 1970s, Ionescu et al. pioneered the use of glutaraldehyde (GA)‐treated bovine pericardium in bioprosthetic cardiac devices for single valve replacement (aortic, mitral, and tricuspid). GA‐treated xenogenic pericardium has since found widespread usage as a cardiovascular tissue substitute for intracardiac and extracardiac repair.^11^, ^12^, ^13^, ^14^, ^15^ However, this treatment led to problems associated with the remaining aldehyde residues, including complications related to inflammation, immunogenicity and cytotoxicity, as well as severe calcification, particularly in the pediatric population.^16^ Various technologies were then evaluated to replace GA and crosslink collagen fibers in pericardium, such as dye‐mediated photo‐oxidation. In 1996, Bianco et al.^17^ reported the implantation of photo‐oxidized pericardial tissue in the mitral position of juvenile sheep, and four of six valves remained free of any calcification while the GA‐fixed valves all exhibited some calcifcation. Photo‐oxidized bovine pericardial tissue has been commercially available since receiving FDA approval in 1999.^18^ Decellularization is another approach to treat xenogeneic or allogenic tissue. In contrast to tissue treated with GA, which reduces immunogenicity by concealing antigenic sites and enhances mechanical strength through the crosslinking of collagen fibers and other proteins, decellularization eradicates cells that can trigger immune rejection while preserving essential mechanical integrity.^19^ These distinct methodologies share the common objective of enhancing stability and minimizing immunogenicity. One type of the decellularized tissue is pericardium, either xenogenic or allogenic. In 1994, Courtman and associates used detergent and enzymatic extraction to yield a decellularized pericardial matrix without sacrificing the mechanical properties of fresh bovine pericardial tissue.^20^ Some of the commercial patches based on bovine and equine pericardium are also pre‐treated by decellularization, as shown in Table 1. Another example of decellularized tissue is porcine small intestinal submucosa extracellular matrix (SIS‐ECM). Matsumoto and colleagues first introduced the use of submucosal tissue for cardiac applications in 1966, when they successfully inserted small bowel grafts into the superior vena cava (SVC) and inferior vena cava (IVC) of dogs for 12 months.^21^ Porcine‐derived SIS‐ECM is now available commercially and has been used extensively.

**TABLE 1:** Representative patch products currently available on the market.

Category	Type	Manufacture/Seller	Commercial name	References
Synthetic Patch	ePTFE	W. L. Gore & Associates	GORE‐TEX Cardiovascular Patch	104
			GOREACUSEAL Cardiovascular Patch	158
	Dacron	Bard Peripheral Vascular, Inc.	Sauvage	43
	Dacron impregnated with collagen	Maquet Getinge Group	Hemacarotid	27
			Hemashield	170
	Polyester‐urethane	B. Braun Melsungen AG	Vascular‐Patch	171
Xenogenic patch	Bovine pericardium	LeMaitre vascular	XenoSure	27
	Bovine pericardium	Edwards lifesciences	Edwards bovine pericardial patch	172
	Bovine pericardium	Baxter	Peri‐guard Pericardium Patch	78
			Vascu‐guard Peripheral Vascular Patch	173
	Bovine pericardium	Abbott	SJM Pericardial Patch	68
	Decellularized bovine pericardium	LeMaitre Vascular, Inc	CardioCel	58, 109, 127
	Decellularized bovine pericardium	Artivion	PhotoFix	18, 51
	Decellularized equine pericardium	Auto Tissue Berlin GmbH	Matrix Patch	59
	Porcine pericardium	Tissue Regenix Group	SurgiPure XD	174
	Porcine pericardium	FOC Medical	SURGIFOC	175
	Porcine pericardium	Vascutek	Vascutek Porcine Pericardial Patch	68
	SIS ECM	CorMatrix	CorPatch	25, 66, 69, 118, 120, 126
	SIS ECM	Aziyo Biologics (distributed via LeMaitre Vascular, Inc in US)	ProxiCor/Tyke	131
Allograft	Human tissue	LifeNet Health	CardioGRAFT (MatraCELL)	176
	Human tissue	Cyrolife	CryoPatch SG (SynerGraft)	177

The third category of CVPs is fabricated through tissue engineering technology. Langer and Vacanti introduced TE in the 1990s to create a substitute with potential for regeneration, remodeling, and growth.^22^ Tissue‐engineered (TE) CVPs can be created from a scaffold of material that is designed to mimic the native ECM. This scaffold material relies mainly on the use of synthetic or biological matrix materials with degradation potential.^23^ Autologous cells can be seeded on these artificial scaffolds to regenerate cardiovascular tissues in vivo. These scaffolds degrade completely in vivo, resulting in autologous tissue formation without foreign material.^24^

### 1.1 Patch requirements and concerns

Most commercial patches are constructed from either polymers or biological materials. However, they are all prone to the same deficiencies, namely unsatisfactory biomechanical response, dehiscence, calcification, thickening, retraction, and degeneration. An ideal patch should be soft (easily deformed under physiological pressure), pliable (easily bent or flexed for better handling), and resistant to tearing, calcification, and shrinkage. It should neither interfere with the patient's growth nor induce adverse remodeling with the formation of scar tissue.^25^ The following factors should be considered when selecting an optimal patch: (1) durability, (2) resistance to infection and late degeneration, (3) Antithrombotic properties, (4) compliance, (5) comfortable handling, and (6) ease of procurement. Patches require a complex combination of physical, mechanical, and biological properties to ensure cost‐effective manufacture and effective, reliable use. TE patches with degradable scaffolds must also degrade and remodel at a proper rate in vivo with no adverse effects. The benefits and drawbacks of different patches will be reviewed to inform the development of a new generation of patches to fulfill these critical requirements.

By providing a comprehensive overview of the current use and prospects of different types of CVPs, focusing on the unique requirements of the pediatric population including the need to adapt to somatic growth as well as higher metabolic rates and more active immune response,^26^ this review article aims to serve as a valuable resource for researchers, engineers, and clinicians interested in harnessing the potential of patch materials for various applications in congenital cardiac surgery to better address the unmet needs in this area and ultimately to improve patient outcomes.

## 2 PATCH TYPES

### 2.1 Synthetic patches

The most widely employed synthetic materials are ePTFE (Gore‐Tex), polyesters (DACRON), and polyurethane (Vascular‐Patch). These patches provide the benefits of being readily available, biostable in vivo, and having a low incidence of early patch rupture.^27^ Other polymeric materials used as patches/scaffolds at the investigative level include polycaprolactone (PCL),^28^ poly(4‐hydroxybutyrate) (P4HB),^29^ poly(lactic‐co‐glycolic acid) (PLGA),^30^ poly(ethylene glycol) (PEG)/PCL,^31^ poly(glycerol sebacate) (PGS),^32^ polycarbonate urethane (PCU),^33^ polydimethylsiloxane‐polyurethane (PDMS‐PU),^34^ along with others. These polymers are frequently utilized either as the biodegradable matrix in TE patches or as a critical biostable polymeric component for potential tissue substitution in cardiovascular grafts.

#### 2.1.1 ePTFE patch

PTFE is a fluoride resin composed of only carbon and fluoride. It is chemically inert and has a very low coefficient of friction and excellent resistance to degradation. ePTFE, the microporous form of PTFE with a fibril distance between 20 and 30 μm, has been used extensively for vascular grafts.^35^

Although ePTFE is generally regarded as a biocompatible material, serious complications have been reported in biomedical applications. For instance, its porous microstructure potentially provides bacteria with an ideal growth environment as it is difficult for eukaryotic immune cells to penetrate it.^36^ Some proteins like albumin and immunoglobulin, can be adsorbed on the surface and provoke a series of foreign body reactions. As a result, thrombosis caused by platelet aggregation on the surface is a common complication associated with ePTFE, supported by multiple research studies.^37^, ^38^, ^39^ Another drawback of ePTFE is that its porosity can interfere with hemostasis, potentially increasing operative time and blood loss. This bleeding can be further exacerbated in cases where patients are anticoagulated intraoperatively. Newer generations of ePTFE commercial patches have included a low‐bleed elastomeric fluoropolymer membrane sandwiched between two ePTFE layers to hinder suture line and cannulation needle bleeding.^40^ Gore‐Tex (Gore Medical, Flagstaff, AZ) cardiovascular patch is the commercial ePTFE product which has been used most extensively for cardiovascular procedures.

#### 2.1.2 Dacron patch

Dacron is a registered trade name for a polyester fiber made by DuPont in the 1950s. It has high tensile strength and elastic modulus, and it is resistant to degradation. Dacron patch aortoplasty has been previously used for aortic coarctation repair. However, problems such as late aneurysm formation have limited its use. Attempts to improve Dacron's performance include the incorporation of biomolecules, such as collagen‐impregnated Dacron.^41^ This version is more useful in vessel replacement and does not require pre‐clotting before implantation. It also does not have the problem of “needle hole” bleeding.^42^ However, the collagen‐impregnated Dacron patch also has a higher incidence of perioperative stroke, thrombosis, and early restenosis compared to the PTFE patch.^43^

### 2.2 Biological patch

The term “biological patch” in the present review includes xenografts, allografts, and autografts. Xenogenic patches, including pericardium‐based patches and SIS‐ECM based patches, are commonly employed as patch prosthetics in research and clinical applications. Some currently available patches on the market are listed in Table 1. Allogenic and autogenic patches have numerous established clinical uses, for example; GA‐treated autologous pericardium patches were used for valve repair,^44^, ^45^ however these patches have relatively limited sources, and autologous pericardium especially is not always available intraoperatively.

#### 2.2.1 Xenogenic‐pericardial patch

Xenogenic pericardium has been applied in cardiovascular surgery since the 1970s. Generally, the pericardium needs to be treated to eliminate antigenicity and offer the patches with tensile strength and maneuverability.^46^, ^47^ Untreated pericardium is prone to retraction and aneurysmal dilation at sites with high intraluminal pressure. Glutaraldehyde has become the standard processing agent, as it forms cross‐links to improve mechanical and immunogenic performance. However, this treatment has major drawbacks. It is cytotoxic and increases the risk of calcification and thickening.^48^ Unexpected calcification and deformation of implanted patches can result in rupture and paravalvular leak if used for valve repair, or false aneurysm formation if used for ventricular repair. The incidence of valve failure due to pericardium calcification was about 10% to 20% at 10 years in adults and 40 to 50% at 4 years in children.^49^

To overcome those GA‐related problems, different technologies were involved to improve the biocompatibility and biostability of treated xenogenic pericardium. A completely non‐GA, dye mediated photo‐oxidation process has been employed to preserve bovine pericardial tissue. This preservation method relies on methylene blue as a photosensitizer and visible light to create new cross‐links in the collagenous substrate material.^50^ This process results in tissue that is biostable, easy to handle, and has a low incidence of calcification, as demonstrated in a series of in vivo tests.^17^ This type of patch has been commercialized as PhotoFix (Artivion, formerly CryoLife, Inc., Kennesaw GA). Although it has been claimed to be non‐immunogenic and relatively resistant to calcification, Majeed and associates reported that compared to GA‐preserved autologous pericardium, PhotoFix was associated with relatively more inflammation and tissue degeneration when used as a tissue substitute during pediatric heart surgery.^51^

Decellularization is another important step to achieve a biocompatible xenogenic patch because this procedure removes the cell antigenicity while preserving ECM with the desired microstructure and biomechanical properties.^52^ The ECM is isolated through the application of physical, chemical, and/or enzymatic treatments to produce a natural scaffold.^53^ Through biochemical and biomechanical cues, this ECM can be recellularized to create functional tissue.^19^ There have been reports of decellularized bovine, equine, and porcine pericardial patches implanted in sheep models of valve reconstruction, with controlled healing, little calcification, and excellent hemodynamic performance.^54^, ^55^, ^56^ An anti‐calcification process (ADAPT) which includes decellularization and modified GA fixation for bovine pericardium tissue was investigated initially by Australian researchers.^57^ The ADAPT process decellularizes tissue by removing lipids, all cells and cell remnants, nucleic acids, and α‐Gal epitopes from bovine pericardium. A modified monomeric GA at a low concentration was still necessary to achieve crosslinking, ensuring the maintenance of natural strength and elasticity, as well as eliminating calcification binding sites and cytotoxicity by detoxification.^57^ The bovine pericardium scaffold treated by ADAPT was commercialized as CardioCel (LeMaitre Vascular, Inc, Burlington, MA, acquired CardioCel from ADMEDUS Pty Ltd) and widely used for the repair of a variety of CHDs, from closing septal defects to augmenting the arch and pulmonary arteries (PAs).^58^ It is noteworthy that a recent study led by Weixler and her team found that when decellularized equine pericardium patches were used for pulmonary artery augmentation, there were higher rates of reoperation compared to the same type of patch used in other locations.^59^ The reoperation/reintervention rates were similar to those observed with other xenogenic materials as reported in other articles, occurring most frequently after PA augmentation.^59^ This finding indicates that the choice of using decellularized pericardium patches for specific applications should be made with caution, taking into consideration the applied location and complex environment.

#### 2.2.2 Xenogenic‐SIS‐ECM patch

Decellularized SIS‐ECM has also been used as a CVP. SIS is a biological biomaterial obtained after removing the mucosa, serosa, and muscle layers from the small intestine of pigs. The advantages of SIS include low immunogenicity, high mechanical strength, and host cell recruitment.^60^ As an acellular bioscaffold, ECM application allows for native cell ingrowth and can be transformed into native tissue in vivo.^61^, ^62^ Porcine SIS‐ECM, is a capable scaffold for the repair and reconstitution of vascular and valvular tissues.

Animal studies have demonstrated promising results for SIS‐ECM patches. Boni et al.^63^ utilized SIS‐ECM for pulmonary artery reconstruction in a lamb model and demonstrated endothelialization with no signs of stenosis or aneurysm formation and importantly confirmed its remodeling and growth potential. Due to positive preclinical performance, this type of material has been manufactured as several commercial products in different generations, such as CorMatrix and CorPatch (CorMatrix Cardiovascular Inc., Atlanta, GA), ProxiCor and Tyke (Elutia Inc., formerly derived from CorMatrix Cardiovascular, Silver Spring, MD). These patches have similar processing procedures and the core component of SIS‐ECM, and they have demonstrated several inherent advantages such as easy handling, effective hemostasis, and low cost^(p400)^.^64^ However, the performance of SIS‐ECM patches can be variable and is particularly dependent on the implantation site. The working environment and mechanical loading are essential to functionalize the SIS‐ECM patch and contribute to its remodeling. For example, when used for atrial and ventricular septal defect closure, these patches have excellent outcomes with few patch‐related complications, as shown in multiple research studies.^65^, ^66^, ^67^ However, it has been seen that at high‐pressure intracardiac sites such as the aortic valve, complications were more likely to occur.^68^, ^69^ It is noteworthy that tricuspid valve reconstruction using a cylinder created from a CorMatrix patch has shown promising results both in sheep and in clinical use.^62^, ^70^, ^71^ And SIS‐ECM based tricuspid valve prostheses are also under investigation, indicating SIS‐ECM could also be a good valve substitution material for tricuspid valves.^72^

#### 2.2.3 Allogenic patch

The use of allograft/homograft for tissue repair has been growing significantly.^73^ Cryopreserved human allografts are used extensively in cardiac surgery; however, they can induce a marked human leukocyte antigen (HLA) alloantibody response with deleterious effects on allograft function.^74^ Therefore, decellularized cryopreserved allograft technology has been introduced to theoretically eliminate this immune response, as well as allow cell in‐growth and improved durability. Decellularization protocols of human pericardium have been reported with the aim to avoid immunogenic reactions in the recipient.^75^ Wollmann et al.^76^ reported using sodium dodecyl sulphate and ethylenediaminetetraacetic acid to remove cells in allograft without affecting the biomechanical properties. However, clinical data on the usage of decellularized human pericardium in patients are still lacking. On the other hand, decellularized allograft pulmonary artery (PA) patches have been commercialized, such as CryoPatch SG (Artivion, Kennesaw, GA) PA patches processed by SynerGraft technology and CardioGRAFT series (LifeNet Health, Virginia Beach, VA) of PA patches fabricated by MatrACELL technology. They are approved by the FDA and used in research and clinical applications. Hawkins et al.^77^ compared antibody response between standard (no decellularization steps) and decellularized allograft patches (CryoValve SG) and found that the latter elicited significantly lower levels of antibody formation and slower rates of degeneration.

#### 2.2.4 Autogenic patch

Autologous pericardium has been widely used as a patch material in cardiovascular procedures. It is inexpensive and easily modifiable. The main advantage compared to other patches is the absence of an immune reaction. It is resistant to infection and has good hemodynamic properties (matching compliance and stiffness, etc.), with low rates of thrombosis and hemolysis. Jiang et al.^78^ evaluated the performance of autologous and xenogenic pericardium in a rabbit model and demonstrated that the former caused less inflammation and immune response with greater resistance to calcification. Although autologous pericardium is inexpensive and has low immune reaction, it has become less popular over time. The main limitations include its limited availability, the complexity of harvesting pericardium if it has been opened in a previous cardiac surgery, the inability to close the pericardium at the end of the surgery, the difficulties in manipulating the autologous patch during usage due to its tendency to shrink, calcify, and form aneurysms.^79^

Another autogenic patch option is autologous vascular patch. This type of patch is harvested from the patient's own vein or artery, and thus can be used directly as the vessel closure and conduits, reducing the risk of immunological rejection and complications. Yang's team presented their work including 24 pediatric patients who underwent aortic arch reconstruction with an autologous vascular patch, harvested from main pulmonary artery, left and right subclavian artery.^80^ They reported no patients showed later hypertension in the 10‐year follow‐up period. Thus, the autologous vascular patch was strongly recommended by their team to augment the lesser curvature in arch reconstruction. In addition, autologous ascending aortic wall has been used as a leaflet replacement material during aortic valve repair since 1959.^81^ Although it fell out of favor for over 60 years, it has recently gained popularity and has been used by Rankin's team.^82^ Eight patients have undergone aortic valve repair using autologous ascending aortic wall, two of whom are pediatric patients (16 and 8 years old). The authors reported that all patients had grade 3 to 4 aortic insufficiency (AI) preoperatively, and each achieved no AI after repair, with all patients recovering uneventfully. It is noted, however, that both pediatric patients required reoperation for unspecified reasons. This raises the question of whether using autologous aorta for valve repair might be less durable in pediatric patients compared to adults. More experience and follow‐up will be necessary to fully assess this approach, especially in the pediatric population.

### 2.3 Tissue‐engineered patches

It is important to note that some researchers also classify decellularized xenografts as Tissue‐engineered (TE) patches, which are derived from different species but retain the ECM framework after special treatment, like ADAPT, to acquire acellular framework supporting host cell infiltration and tissue regeneration. In this review, TE patches will be specifically referred as man‐made, biodegradable scaffolds that mimic the native ECM, offer mechanical support and cell delivery, manipulate cell ingrowth, and eventually degrade with neo‐tissue formation.^83^, ^84^

These TE patches aim to provide an extracellular matrix that mimics microenvironmental cues, favoring the integration and remodeling of the implant.^85^ This matrix provides mechanical support and attracts host cells, including endothelial cells, smooth muscle cells, and fibroblasts, into the construct.^86^ When the patch interacts with blood components, it triggers a controlled inflammatory response, leading to the recruitment of various immune cells and macrophage infiltration. The infiltrated cells secrete cytokines and growth factors that attract additional immune cells, as well as tissue cells originating from surrounding tissue and/or circulating progenitor cells, to promote tissue remodeling.^87^ Endothelial cells line the interface between the blood and the scaffold, while activated myofibroblasts infiltrate the scaffold to synthesize extracellular matrix, creating a new scaffold for tissue growth. If the implanted patch is made of biodegradable material, the ECM matrix may degrade over time to (1) provide space for native tissue ingrowth and (2) decrease pro‐inflammatory stimuli, eventually leading to the resolution of inflammation. Ideally, the patch should integrate seamlessly with the surrounding tissue, providing mechanical support and promoting tissue regeneration. The success of the integration process is ultimately determined by the balance between these cellular and molecular events.

**FIGURE 2:**
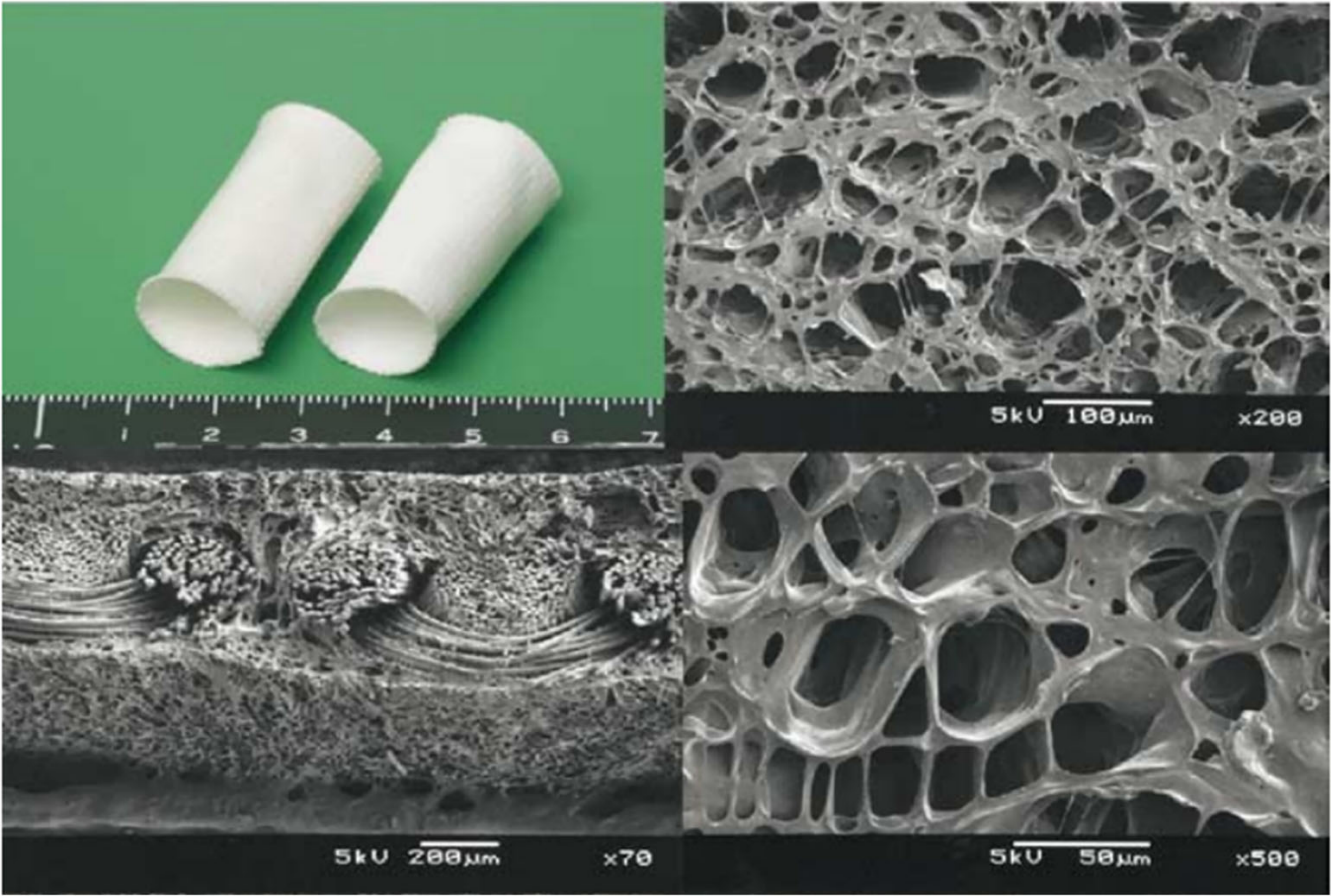
Macroscopic appearance and Scanning Electron Microscopy (SEM) images of biodegradable polymeric scaffolds. Top left: A scaffold with a diameter of 18 mm, fabricated from a woven matrix of PCL and PLA (50:50) and reinforced with PGA mesh. SEM images reveal the scaffold's internal morphological structure. Scale bars correspond to 200 μm (bottom left), 100 μm (top right), and 50 μm (bottom right). Reprinted from Reference [30], copyright (2005), with permission from Elsevier.

Generally, these biodegradable scaffolds can be made of synthetic degradable polymer matrices, or chemically crosslinked natural materials, or composite (hybrid) components to synergistically augment desired cellular and tissue responses (Figure 1).^88^, ^89^ This type of CVP is commonly fabricated by various techniques, such as solution casting, electrospinning, and 3D printing and has been evaluated via a series of in vitro and in vivo characterization methods.^90^, ^91^ TE patches can be modified by (1) manipulating scaffold properties including biocompatibility, biomechanical properties, and rate of degradation, (2) seeding the scaffolds with different cell sources such as endothelial and smooth muscle cells,^92^ bone marrow cells,^93^ embryonic stem cells^94^ and induced pluripotent cells,^91^ and (3) applying the appropriate environmental cues (growth factors, physical stimulation) to encourage tissue in‐growth. The most important and promising advantage is that they provide a platform for living autologous tissues to grow, remodel, rebuild, and repair.^95^ Sugiura et al.^96^ utilized freeze‐drying and electrospinning technology to fabricate a PLCL scaffold composite enhanced with PLLA fibers. These bioresorbable grafts displayed cell infiltration into the scaffold, growth of endothelial cells, and deposition of collagen and elastin in mouse models. Vascular tissue formation was observed when placed in an arterial circulation environment. Masoumi et al.^32^ chose a combination of microfabrication and electrospinning to create an accessible platform for cell infiltration and growth, developing a three‐layered biomimetic scaffold for tissue‐engineered heart valves. The biodegradable matrix, composed of PGS and PCL, performed well in a porcine ex vivo model of heart valve leaflet replacement. Clinically, Shinoka and his team began human trial evaluating the TE grafts in pediatric patients with single ventricle physiology since 2001 and exhibited the satisfactory mid‐term and late‐term results.^30^, ^97^ The mononuclear cell component was collected from autologous bone marrow, then cells were seeded onto a biodegradable scaffold made of PGA and Poly(l‐lactide‐co‐ε‐caprolactone) (PLCL, the copolymer of L‐lactic and ε‐caprolactone) as extracardiac cavopulmonary conduit (Figure 2).^30^ 23 such TE grafts and 19 TE patches were implanted to pediatric patients (median age, 5.5 years) and no thrombotic, stenotic, or obstructive complications were observed (follow‐up 1.3–31.6 months, median 16.7 months). All grafts were patent with no evidence of aneurysm formation or calcification in this mid‐term evaluation.^30^ Subsequently, the same team reported late‐term results of these TE grafts. There was not any graft‐related mortality (mean follow‐up, 5.8 years). Neither aneurysm formation, graft rupture, infection or ectopic calcification were reported, but four patients developed graft stenosis, which lead to graft failure.^97^ Nonetheless, the assessment of TE grafts and patches has been limited to clinical trials, and currently there is not any TE patches made entirely from synthetic and/or biological polymers available on the market. This is likely due to (1) the challenge of inducing immune tolerance of the TE grafts, and (2) a shift in focus from single patches to more complex devices, such as TE blood vessels or valves.^98^ In line with this trend, Xeltis (Eindhoven, the Netherlands) introduced a fully bioabsorbable valved conduit (Xeltis Pulmonary Valved Conduit; XPV) and a pulmonary valve prosthesis (Xplore‐2) for clinical use in children, which have shown promising early clinical outcomes.^99^, ^100^

In summary, each type of patch has its advantages and disadvantages, as shown in Table 2. Patch selection should be guided by individual factors such as surgical procedure, implantation site, and the conditions being treated. In the next section, patch selection and performance will be discussed in detail.

**TABLE 2:** Advantages, drawbacks, and pretreatment of various types of cardiovascular patch.

Patches	Advantages	Drawbacks	Treatment
ePTFE	‐Porous, allows tissue ingrowth ‐Nonimmunogenic ‐Nondegenerative ‐Stable ‐Good biocompatibility ‐Inexpensive	‐Bleeding through needle holes ‐Can be too stiff depending on the application ‐Leading to thrombosis due to lack of a natural endothelial lining	N/A
Dacron	‐Durable ‐Stable ‐Readily available	‐Aggressive inflammatory response ‐Fibrosis ‐Less elastic ‐Compliance mismatch	N/A
Xenograft pericardium	‐Reliable consistency ‐Little bleeding through needle holes ‐Good biocompatibility ‐Compliance similar to native arterial tissue ‐Facilitate cell migration and tissue regeneration	‐Immune reaction ‐Calcification ‐Risk of infection	1. Pre‐treated by Glutaraldehyde 2. Decellularization 3. Photo‐oxidation
SIS‐ECM	‐Biocompatibility ‐Manageability ‐Easy handling ‐Easy hemostasis ‐Potential for remodeling	‐Inflammatory response ‐Fibrosis ‐Uncertainty on remodeling	Decellularization
Allogenic patch	‐Ease of handling ‐Limited immune response ‐No bleeding through needle holes	‐Limited sources	Cryopreserved
Autologous pericardium	‐Available ‐Nonporous ‐No bleeding through needle holes ‐Resist infections ‐Low thrombosis ‐Low hemolysis ‐Low cost ‐No immune response	‐Susceptible to aneurysmal dilatation ‐Retraction ‐Fibrosis ‐Limited quantity ‐Difficult to harvest after prior sternotomy	Fixation with different concentration of glutaraldehyde improves handling characteristics (inexpensive, simple)

## 3 PATCH APPLICATIONS

Many of the most common CHDs require surgical interventions with patches, including structural abnormalities of the great vessels, cardiac septal defects, and valve defects. Generally, the use of patches in congenital cardiovascular surgery falls into broad categories: *vascular repair*, *valvular repair*, *septal defect closure*, and *intracardiac baffle*. Some complex cardiac procedures require the use of patches in more than one of these applications.

### 3.1 Vascular repair

Coarctation of the aorta (CoA) is the sixth most common CHD. The optimal surgical management for CoA is still being formulated, especially in neonates, infants, and young children.^101^ Patch augmentation of the hypoplastic segment was once the preferred surgical technique. However, it has been associated with a high rate of aneurysm formation, re‐coarctation, and late hypertension.^102^, ^103^ Kaya and associates investigated the early and late results of different surgical treatments for CoA in children and adults.^104^ In this study, one in seven patients (14%) who underwent patch aortoplasty with a PTFE or Dacron patch experienced re‐coarctation, whereas one in 13 patients (8%) who underwent end to end anastomosis developed re‐coarctation. Their clinical experience suggested that the most appropriate treatment option in the infantile period is resection and direct end‐to‐end anastomosis because of favorable anatomic conditions in this age group.^104^ Patch aortoplasty may be preferable in older patients, or in young patients with recurrent coarctation or long segment coarctation. Lee and colleagues reviewed 31 patients (range 5–200 days) with CoA and arch hypoplasia who underwent augmentation of the lesser curvature of the aorta with autologous vascular patch harvested from the aortic isthmus, pulmonary artery, subclavian artery, or distal arch.^101^ They hypothesized that these autologous vascular patches would achieve an arch with a more natural shape, avoid acute angulation, extensive mobilization of the aorta, and potential restenosis or late hypertension. No recoarctation was observed during a median follow‐up of 24.8 months and no patient needed reoperation. This outcome could be attributed to achieving a more natural shape of the arch using the autologous vascular patch, which also minimized complications. Autologous pericardium has also been used for aortic arch reconstruction as reported by Bernabei's team.^105^ They reported its use in 39 newborns (range 1–35 days) with an overall incidence of recurrent arch obstruction 28.2% occurred within 5.3 ± 2.59 months. Xenogenic patches as commercially available options, have also been used in CoA repair. Deorsola and associates used CorMatrix to treat a 3‐week‐old male patient with CoA, and while there was no evidence of aortic dilation at 1 year, the patient did develop restenosis 5 months after surgery.^106^ The authors inferred that this restenosis was caused by growth of ductal tissue on the patch. Bovine pericardial patches have also been used in such cases, however, they have been found to be prone to restenosis in infants due to unsatisfactory mechanical properties under high systemic pressure.^58^ Similarly, Vitanova et al.^107^ reported using different patch materials to reconstruct the aortic arch in 145 pediatric patients. They found that xenogenic pericardium (equine, *n* = 28) had a significantly higher rate of recoarctation than allograft (*n* = 87) and autologous pericardium (*n* = 23) and freedom from recoarctation was 30% ± 22%, 85% ± 4% and 86% ± 7% at 2 years, respectively. In a similar comparison study, Beynum et al.^108^ used two different patch materials, homograft (*n* = 26) and CardioCel (*n* = 10) for reconstruction of the aortic arch in neonates and infants. Six (23%) patients in the allograft group and seven (70%) patients in the CardioCel group developed restenosis during the first year, and the median time from operation to first restenosis intervention was 22 weeks for the homograft group, as compared to 14 weeks in the CardioCel group.

In general, for CoA and other types of aortic reconstruction in pediatric patients, especially for neonates, resection and direct anastomosis produces superior outcomes. If a patch is required, an autologous or homograft patch is recommended rather than a xenograft patch.

In addition to repair of CoA, other vascular repairs, such as PA reconstruction, repair of supravalvular aortic stenosis, RVOT repair, and the like, have also been performed with various patch materials.^18^, ^25^, ^109^ Baird et al.^18^ reported a series of 168 patients who underwent PA reconstruction with PhotoFix patch, with overall survival of 92% at 6 years with no deaths attributed to patch failure. However, nine out of 53 (17%) patients who underwent left PA plasty had a reoperation (there were no reported reoperations among patients who underwent main or right PA plasty), and 25 out of 168 (15%) patients who underwent PA patch plasty had a catheter based reintervention. Ebert et al.^110^ compared PA homograft, bovine pericardium, autologous pericardium, and porcine SIS‐ECM patch used in PA reconstruction and found that autologous pericardium, the least expensive material, had the lowest reintervention rate (7.3%) compared to 16.2%, 18.2%, and 18.5% for porcine SIS‐ECM, bovine pericardium, and PA homograft, respectively; however, patch material was not associated with reintervention in multivariable cox regression.

In Tetralogy of Fallot (TOF), a transannular patch is often used to relieve RVOT obstruction, with significant drawback of post‐operative pulmonary valve incompetence. Autologous pericardium has traditionally been used, and there is increasing interest in utilizing new materials to improve postoperative outcomes.^111^ Xenograft pericardium is particularly useful when the patient had a previous procedure and sufficient pericardium is not available, however outcomes are inferior to autologous pericardium due to the host immune response and severe degree of calcification.^112^ Simon et al.^113^ studied TOF repair using a Dacron patch in a transannular patch or a valve sparing RVOT patch depending on pulmonary valve Z‐score and found a 5‐year freedom from reoperation of 95% with no difference between the groups at 10 years (95.6% vs. 91.8%, respectively). The authors also noted that the non‐distensibility of Dacron limits progressive pulmonary insufficiency when used as a transannular patch. Miyazaki et al.^114^ studied ePTFE patches and found no or mild pulmonary insufficiency in 79.6% of patients with 10‐year freedom from reoperation of 92.3%, which makes this a promising strategy for RVOT reconstruction.

### 3.2 Valvular repair

Performing aortic valve repair, rather than replacement, in pediatric populations is effective in postponing reintervention.^44^ CVPs have a wide application in valve repair. Aortic cusp extension valvuloplasty (ACEV) utilizes GA‐treated autologous pericardium to treat aortic stenosis (AS) and aortic insufficiency (AI).^115^ ACEV has been increasingly used for children and adolescents with excellent survival and good long‐term outcomes.^115^ Freedom from a second aortic valvuloplasty and aortic valve replacement at 18 years was 82.1% ± 4.2% and 60.0% ± 7.2%, respectively. Similarly, this technique has been applied for rheumatic aortic regurgitation with freedom from reoperation at 10 and 20 years of 96.7% and 66.6%, respectively.^116^ The long‐term results of using GA‐treated autologous pericardial patches showed acceptable durability and a low incidence of thromboembolic events or bleeding.

CorMatrix has also been used for aortic valve leaflet extension, however, with conflicting results. Quarti et al.^25^ published their series including nine patients who underwent valve reconstruction using CorMatrix. At a mean follow‐up of 13.2 months, there were no patch‐related complications and no cases of patch failure or calcification. On the other hand, Hofmann and associates presented their experience using CorMatrix for aortic valve repair in six pediatric patients, in which significant valve insufficiency developed in 83.3% (*n* = 5) patients during the post‐operative period (119–441 days postoperatively).^117^ The expected process of seeding/migration and remodeling were not observed in explanted specimens. Only a migration of inflammatory cells was identified, which induced structural and functional changes. Woo et al.^118^ also demonstrated similar results such that there was not any evidence of constructive remodeling found in 12 explanted CorMatrix samples used in pediatric cardiac reconstruction. One retrospective study reported by Hu et al.^119^ found as well that patients who received CorMatrix patches had a significantly decreased rate of freedom from reoperation (12.5%) compared to those who were treated with autologous pericardium patches (62.5%), due to the development of severe aortic regurgitation at 5‐year follow‐up.

**FIGURE 3:**
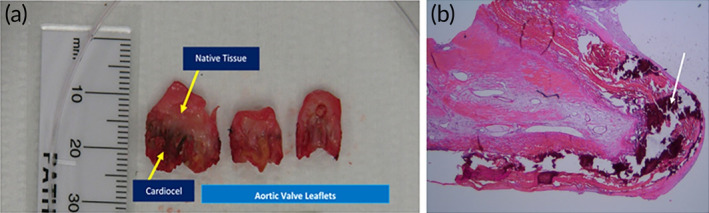
(a) Gross appearance of explanted aortic valve leaflets. (b) Microscopic appearance (10× magnification) after hematoxylin and eosin staining of an explanted CardioCel patch. The white arrow points to the foci of calcification. Adapted with permission from Reference [124]. Copyright The Authors (2021), with permission from SAGE.

Other patches, such as GA‐fixed bovine pericardium or ePTFE, have shown disappointing long‐term outcomes when used for cardiac valve reconstruction, with major concerns related to limited durability and patch fibrosis, degeneration, thickening, retraction and calcification.^120^ Advanced pretreated technologies, like ADAPT process for CardioCel, or non‐aldehyde, dye mediated photo‐oxidation process for PhotoFix, have been applied to reduce the complications arising from GA. Chivers and coworkers presented a series of work where five children and young adults who underwent the Ozaki procedure (aortic valve neo‐cuspidization^121^, ^122^) with CardioCel.^123^ After a mean follow‐up of 29.6 months, two patients had valve related complications: one had severe aortic stenosis due to fibrosis and calcification and the other one had aortic stenosis suspected to be secondary to inflammation and fibrosis of this neo‐aortic leaflets. In another case reported by Patukale et al.,^124^ an 11‐year‐old child underwent aortic valve repair including augmentation of all three leaflets with CardioCel. All three CardioCel leaflet extensions were calcified, rigid, and immobile at explantation after 38 months, while the native aortic leaflet remained supple (Figure 3a). Microscopic examination of the valve displayed thickening of the native tissue with myxoid stromal degeneration and fibrosis. CardioCel exhibited patchy neo‐intimal fibrosis, surface endothelialization, and extensive nodular calcification (Figure 3b). The authors concluded that the patient developed progressive aortic stenosis because the mechanical properties of the repaired patch differed from those of the native valve, leading to a differential response to shear stress in the repaired leaflet, which accelerated the degeneration of the CardioCel patches. Although the use of a Dacron tube graft for aortic root replacement in a pediatric patient could have contributed to valvular degeneration and stenosis, the differential response to stress of the CardioCel patch compared with the native aortic leaflet, including significant calcification in the patch, seems to be the dominant reason for the failure of the aortic valve repair, as indicated in the authors' discussion of their results.^124^ Myers' team demonstrated that PhotoFix (*n* = 16) has better durability than GA‐fixed bovine pericardium (*n* = 9), and its performance can be as good as fresh autologous pericardium when used for cusp extension.^125^ This outstanding performance was attributed to pre‐treatment via dye‐mediated photo‐oxidation rather than GA chemical cross‐linking, thereby rendering PhotoFix patches more biocompatible and less prone to calcification.

CVPs can also be used for repair of the pulmonary valve,^120^ mitral valve,^126^ and tricuspid valve.^127^ Patukale et al.^128^ reported transannular patch and PV leaflet augmentation for TOF or DORV with PV stenosis using ePTFE in 76 patients and GA‐treated autologous pericardium in 46 patients. The median age at repair was 8.9 months. There was no mortality in a median follow up of 6.25 years and greater freedom from moderate or severe pulmonary regurgitation at last follow up in patients with repairs using ePTFE (*p* < 0.001). In a study of 60 patients who underwent PV transannular patch with monocuspid valve reconstruction using PTFE (*n* = 30) or autologous pericardium (*n* = 30), Sayyed et al.^129^ found no statistically significant difference in outcomes between PTFE and autologous pericardium groups at 3 years.

Mitral valve (MV) repair has been reported using both autologous pericardium and SIS‐ECM.^130^ Arbona et al.^131^ reported 29 patients who underwent repair of the MV and/or annulus using SIS‐ECM. Of the 19 with that underwent valve repairs, 90% had freedom from more than mild mitral regurgitation after 6 years.

SIS‐ECM has also been used in tricuspid valve (TV) repair. Gerdisch et al.^70^ studied the use of a cylinder made from a CorMatrix for TV repair in 19 endocarditis patients. There were no deaths, heart block, stroke, or prosthesis mismatch on 1–18 months of follow up. Three patients underwent reoperation due to suture tearing through papillary muscle or conduit disruption, and the remaining 16 patients had no more than mild tricuspid regurgitation. In the pediatric population, one case of SIS‐ECM TV repair reported by Glenn et al.^71^ showed mild to moderate tricuspid regurgitation, and no tricuspid stenosis, and no calcification at 5 years.

Considering that patches will be implanted in locations with variable environmental pressures and flow shear rate when used as valve repair, the chance of deterioration and failure can be higher than in other applications. For instance, Neethling et al.^132^ found that CardioCel provided stable repair when used in non‐valvular positions. They applied these patches to the septal, systemic, and pulmonary vascular positions in 30 children, and after 12‐month follow‐up, they showed no signs of degeneration or calcification. Conversely, in a report of 101 patients in whom CardioCel was used in the septal, valvular, and arterial positions, there were four graft failures in the aortic position and one in the aortic valve position were observed over a median follow‐up of 212 days.^58^ The authors reported that CardioCel suffered from thick neointimal proliferation, which could potentially obstruct the aortic position. Similarly, Chivers et al.^123^ advised caution when using the Ozaki procedure with CardioCel in pediatric and young adult patients, as two out of five patients (40%) developed valve‐related complications. Although this is a small series from a single institution, the accelerated structural deterioration observed warrants caution when using this patch in the aortic position in young populations. In a recent study, Patukale et al.^133^ evaluated the performance of CardioCel in cardiac surgery from January 2013 to December 2020 and also warned that the use of CardioCel in aortic valves and aortic arches requires caution. The narrow lumen and high blood velocity in these areas can lead to higher shear stress and potential propagation of calcification and deterioration. Thus, due to the greater stress placed on CVPs used in a valvular position, less advantageous outcomes may be expected in valve repair applications, and the use of CVPs for valve repair warrants caution.

### 3.3 Defect closure

**FIGURE 4:**
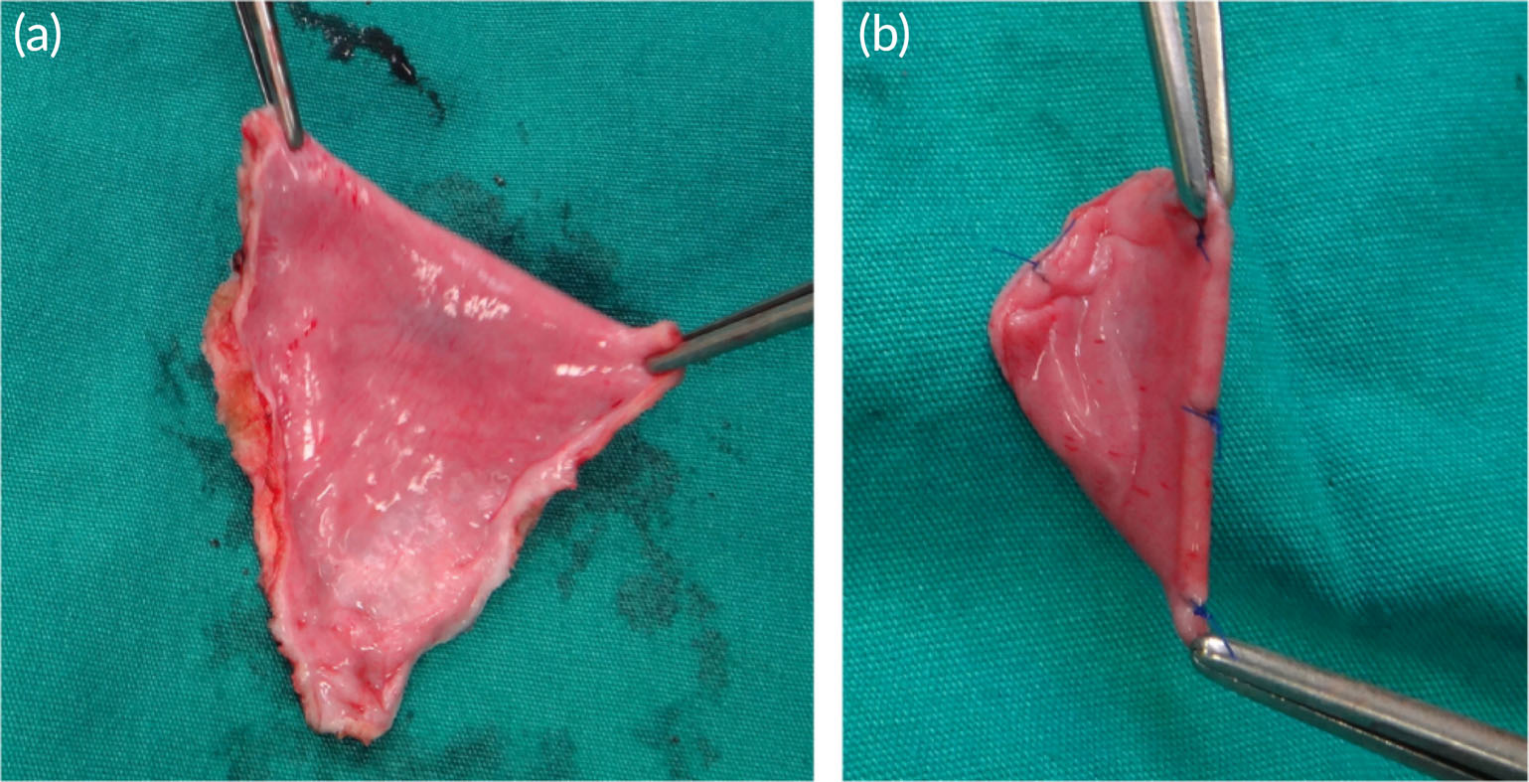
Schematic of a double‐layer autologous pericardial patch for ASD closure. (a) Preparation of the autologous pericardial patch; (b) Sewing the edges to expose the smooth surface. Reprinted from Reference [135], copyright (2014), with permission from Elsevier.

CVPs are also used in the repair of septal defects including atrial septal defects (ASD) and ventricular septal defects (VSD). Hopkins and colleagues reported using fresh autologous pericardium, GA‐treated bovine pericardium, or photo‐oxidized bovine pericardium to close ASDs in 129 children with excellent outcomes: no patient sustained a stroke, severe bleeding or required a transfusion.^134^ Although pericardium is a suitable material for ASD closure, the external surface of the pericardium is irregular and may be a nidus for thrombus formation. To avoid any thromboembolic complications, Aksüt and colleagues used a folded double‐layer of fresh pericardium for ASD closure (Figure 4).^135^ This solution avoided the need for chemical pre‐treatments such as GA fixation and also exposed the smooth surface outside to reduce the thrombus formation. Witt et al.^66^ used CorMatrix for septal defects (ASD *n* = 11 and VSD *n* = 2) in pediatric patients with a mean follow‐up of 411 days with good performance. They concluded that, compared to other applications like vascular augmentation and valve reconstruction, septal defect patching appears to be the safest and most durable application of this product. More generally, CVP performance for closure of septal defects is more durable and reliable than other applications, likely due to the lower stress on patches in these locations.

### 3.4 Intracardiac baffle

Patches can also be used as a baffle to redirect blood flow in patients with CHDs. For example in hypoplastic left heart syndrome (HLHS), the patch can be employed as an internal Fontan baffle to create a path in the right atrium for linking the IVC and PA. PTFE, bovine pericardium, and autologous pericardium are available for baffle creation, and there are no published reports of severe complications or reoperations due to baffle failures.^136^, ^137^, ^138^ CorMatrix has been used by Nelson et al.^69^ as a hemi‐Fontan baffle in 12 patients. After a median follow‐up of 21 months, CorMatrix performed well as a baffle, remaining pliable and with minimal calcification, but all specimens showed evidence of fibrosis, chronic inflammation, and foreign body giant cell reaction without native cell ingrowth. The authors concluded that CorMatrix needs to be evaluated further at different implantation sites because the mechanical loading contributed to a microenvironment which impacts remodeling in the patch.

The Mustard procedure, an atrial switch technique employing intracardiac baffles to reroute blood flow from the SVC and IVC to the left atrium, and pulmonary veins to the right atrium, was initially developed for treating dextro‐Transposition of the Great Arterie (d‐TGA).^139^ Although the arterial switch operation has now largely replaced it for d‐TGA treatment, the Mustard procedure remains indispensable in managing other complex cardiac anomalies, such as in the double switch procedure for congenitally corrected transposition of the great arteries (ccTGA), and patch‐related complications of prior Mustard procedure patients also occur in clinical practice.^140^ In one study 75 patients who underwent the Mustard procedure using autologous pericardium as the patch material, 13 required reoperation for baffle dysfunction, most commonly stenosis of the baffle/SVC junction due to fibrosis and shrinkage of the autologous pericardium baffle.^141^ This complication was treated with baffle enlargement using a Dacron patch and the authors report complete relief of symptoms after reoperation. Another study of 33 patients who underwent the Mustard procedure had 11 patients who developed baffle obstruction and found that this complication was associated with the use of Dacron for the baffle material, with a median time to reintervention of 8 months.^142^ They treated this complication by using a PTFE patch to enlarge the baffle and reported no subsequent recurrence.

The Rastelli procedure consists of an intracardiac baffle that redirects flow from the left ventricle to the aorta through the VSD and an external valved conduit that connects the right ventricle (RV) to the PA. This procedure was initially conceived to be used in patients with d‐TGA, VSD, and pulmonary stenosis, but its use has been expanded to patients with truncus arteriosus, pulmonary atresia with VSD, and double outlet right ventricle.^143^ Brown et al.^144^ studied long term outcomes of the Rastelli procedure using autologous pericardium or PTFE for the VSD to aorta baffle. There were no operative deaths and three late deaths at a median of 8.6 years of follow up. None of the deaths were attributed to patch failure. Kreutzer et al.^145^ studied 101 patients who underwent the Rastelli procedure using a Dacron patch (*n* = 89), PTFE (*n* = 2), and autologous pericardium (*n* = 8) (patch material was not described for two patients). Early mortality was 7% with three deaths attributed to left ventricular outflow tract obstruction (LVOTO). Late mortality was 17% at a median follow up of 8.5 years. In this series, use of a pericardial baffle was associated with decreased time to late death on multivariate analysis (*p* = 0.01).

## 4 PATCH PERFORMANCE

### 4.1 Durability

Patch properties can change over time due to reactions with blood, mechanical loading, and biodegradation. The durability of a patch is therefore essential to ensure the patch's long term efficacy. The cases reviewed below include short‐term or long‐term evaluation of the patch's clinical performance. However, it is essential to consider the precise application of a patch when discussing its durability. Even with the same pre‐treatment, the exact site of implantation will impact its overall performance. Therefore, durability will be qualitatively described according to the authors' statements.

Kumar et al.^146^ evaluated ePTFE patches which were used in 171 pediatric patients (median 1.1 years old) to reconstruct the RVOT. They reported excellent short‐term and mid‐term performance after 10.9 ± 5.8 years mean follow‐up, particularly in TOF with non‐salvageable pulmonary valve or pulmonary atresia/ventricular septal defect (PA‐VSD). The authors extolled the ready availability and durability of ePTFE patches compared to selected pulmonary allografts.

Gluck's team reports long‐term outcomes using a GA‐treated, cryopreserved homograft pericardium patch in neonates, infants, children, and young adults undergoing congenital cardiac surgery.^147^ 276 patches were implanted in 134 consecutive patients with the median age at implantation of 1.47 years. Most of the patches were used in vascular repair and septal repair (*n* = 124 for PA repair, *n* = 57 for aorta repair and *n* = 49 for septal repair), three were used for valve repairs (*n* = 1 for AV, PV and MV, respectively). The 10‐year freedom from patch‐related reoperation and catheter intervention rates were 88.5% and 86.9%, respectively. Overall patch failure–free survival was 85.8% and 79.0% at 5 and 10 years, respectively.

Neethling's team reported the use of 34 CardioCel patches in pediatric patients (*n* = 30) with excellent medium to long‐term durability and median follow‐up of 7.2 years. It's noted that most of the patches were used in septal closures (VSD, *n* = 20; AVSD, *n* = 3; ASD, *n* = 3) and no valvular repair cases reported.^148^ Bell's team reported implanting 501 CardioCel patches in 377 pediatric patients.^149^ Medium term durability in ASD/VSD (*n* = 183), vascular repair/reconstruction (*n* = 229), and valve repair (*n* = 30) was good, with an overall re‐intervention‐free survival was 96% at 5 years.

Woo et al.^118^ used CorMatrix in different pediatric cardiac reconstruction procedures (*n* = 532). After a mean follow‐up time of 518.6 days, only 12 specimens, including four valves and eight septal/outflow/conduit patches, were explanted and only six cases demonstrated patch failure prior to surgery (*n* = 6, 1%), suggesting the CorMatrix' durable performance without clinical signs of patch failure in most cases. However, there was no histologic evidence that CorMatrix acts as a scaffold for reconstitution of the native cardiovascular structures due to the presence of fibrosis and myxoid degenerative changes. Significant degeneration was seen in a majority (9/12) of explanted outflow/septal/valvuloplasty specimens, suggesting that high flow states may predispose CorMatrix to increased degradation. In another case, Fraint and her team compare CorMatrix (*n* = 48) with other types of patch materials (*n* = 84, autologous pericardium and PA tissue; *n* = 19, xenograft pericardium; *n* = 65, homograft and *n* = 5, Gore‐Tex) used in pediatric patients undergoing PA augmentation.^150^ They found that median time to reintervention for all patients was 1099 days and there was no significant difference in reintervention‐free survival between CorMatrix and other patch materials (*p* = 0.18). However, Padalino and colleagues reported the rate of catheter‐based reintervention was higher when using CorMatrix compared to other materials for PA reconstruction in infants and thus did not suggest using CorMatrix for PA‐plasty.^67^ They also found a high rate of functional failure, with a progression to regurgitation or stenosis in 21 out of 38 patients (55.2%) who underwent semilunar valve repair with CorMatrix. Clearly, controversy still exists regarding the use of CorMatrix in the repair of CHDs and more long‐term studies are required to determine which applications are best suited to this patch material.

### 4.2 Biocompatibility

Cardiovascular patches need to consider biocompatibility to avoid any adverse effects. The most important qualities for biocompatibility are anti‐thrombogenicity, anti‐calcification, and hemostasis.^151^

Thrombus formation has been seen relatively more frequently in synthetic patches such as ePTFE and Dacron due to the nature of the synthetic polymer, which can elicit an immune response and cause a foreign body reaction, ultimately resulting in the formation of thrombus. These patches also have the potential for hemolysis and platelet activation associated with non‐physiologic high shear stress.^152^ The rupture of erythrocytes releases adenosine diphosphate (ADP), a platelet activation factor leading to more platelet aggregation on the surface and ultimately resulting in thrombus formation.^153^ Thrombosis is rarely reported as a major problem with biologic patches, either acutely or chronically.^154^ However, calcification has been widely reported as one of the leading causes of cardiac biomaterial failure, especially with xenograft patches.

Calcification is one of the major drawbacks of biological materials and has been linked to GA treatment of biological materials.^155^ GA can form cross‐links to increase patch strength and reduce antigenicity; however, it brings the potential for severe calcification. Moore et al.^48^ hypothesized that this GA‐mediated calcification was due to (1) Mechanical factors associated with tissue stiffness and stress, (2) The cytotoxic properties of GA leading to the deposition of cellular debris which provide nidus points for calcification, and (3) GA's chemical structure attracting calcium ions. Based on this phenomenon, some bovine pericardium have been commercially modified to use a very low concentration of GA (ADAPT process for CardioCel), or avoid GA pretreatment (photo‐oxidation process for PhotoFix) altogether. Reports aforementioned using CardioCel have demonstrated good outcomes regarding anti‐calcification.^58^, ^109^, ^127^ PhotoFix is also demonstrated to have acceptable outcomes with resistance to calcification.^18^, ^51^ However, the group from Boston Children's hospital found that using PhotoFix in an area of high pressure and flexion (ventricular septum and left ventricle) could lead to higher rates of calcification. In contrast, PhotoFix exposed to lower stresses showed little or no calcification, indicating the role of implanted environment patch performance.^18^, ^51^ CorMatrix, on the other hand, does not undergo GA treatment, and thus it exhibits resistance to calcification. No calcification was detected either at reoperation or on medium‐term histological analysis in 103 patients who underwent CHD surgeries.^67^ As reported elsewhere, CorMatrix exhibits good anti‐calcification performance in both small and large studies and may be resistant to the degenerative calcification process.^25^, ^118^

Hemostasis has rarely been reported as an issue with most CVPs, except for the synthetic patches. The porosity and limited elasticity of ePTFE patches leads to prolonged suture‐line bleeding, resulting in longer operation time and significant blood loss. Rittoo and colleagues applied Gelatine–resorcine–formol (GRF) glue as a sealant for ePTFE patch suture lines and showed that hemostasis time was decreased from 20 to 11 min and average blood loss was also decreased from 332 to 224 ml, compared to the group without GRF glue.^156^ Besides applying GRF glue, ePTFE has been modified further by Gore in the commercial product ACUSEAL to create an advanced elastomeric fluoropolymer that can seal around penetrating sutures and improve hemostatic performance.^157^ According to Stone et al.,^158^ the ACUSEAL patch can decrease mean hemostasis time to 4.9 min, comparable to the hemostasis time of bovine pericardium (3.09 min), with low rates of stroke and restenosis. On the other hand, Dacron has also been impregnated with collagen in the commercial product HEMASHIELD to achieve better hemostasis. AbuRahma et al.^159^ compared ACUSEAL and HEMASHIELD in a randomized trial for carotid endarterectomy closure, and found that the mean hemostasis time for the ACUSEAL and Hemashield patches were 5.1 and 3.7 min, respectively (*p* = 0.01). Both patches have short hemostasis times and similar early restenosis rate.

### 4.3 Compliance and usability

Compliance is one of the most fundamental properties to mimic because a patch implanted in a vessel or the heart will undergo various mechanical deformations (tension, flexure, and compression) depending on the implanted site. At the same time, this patch must have proper, matching compliance that does not alter blood flow and maintains normal deformation like functional native tissue. The ultimate goal is that a patch can mimic the compliance of native tissue. Generally, biological patches have better compliance than synthetic ones. Autologous pericardium is known for its excellent compliance (matching mechanical properties) and hemodynamics. Xenografts like bovine pericardium or ECM patches perform hemodynamically similarly to native tissue and contain the biological cues necessary for biointegration.^23^ The synthetic patches may perform more like a foreign body inherently, so biostability and biocompatibility need to be optimized in these products.

In terms of usability, untreated pericardium patches may have fewer desirable characteristics and may not meet certain criteria such as being readily available or easy to handle in surgical procedures. Those non‐preserved patches such as fresh autologous pericardium may curl up after harvesting, which makes them hard to handle in surgery. GA‐treated autologous patches also need to be harvested first, affixed to the plate to prevent wrinkling, cleaned of any adipose tissue, then finally immersed in GA, taking up valuable operative time. From this point of view, ePTFE and any other commercially available patches which better retain their shape, are ready to use and easier to handle.

### 4.4 Mechanical properties

Few clinical reports have investigated mechanical properties; however, it has been noted and reported that the mechanical properties are strongly associated with the patch's overall performance: patches used for ventricular and vascular repair must have the strength to tolerate peak systolic pressure, and patches used for aortic valve repair must exhibit significant anisotropic behaviors. If the mechanical performance of the patch does not match the adjacent tissue, negative outcomes may include thrombogenicity, calcification, and degeneration.^160^, ^161^, ^162^, ^163^ Mismatched mechanical properties between patches and surrounding tissue will lead patch fibrosis and excessive stiffness of the patches can lead to increased inflammation and loss of surrounding tissue's elasticity.^164^

Smaill et al.^165^ have performed an in vitro study to determine the effects of Dacron patch angioplasty on aortic geometry and strain adjacent to the patch. They found that the development of aneurysms resulted from the combined effects of patch geometry, dimensions, and material properties on the distribution of stress and strain in the adjacent aortic wall. Mismatched mechanical performance of the aorta and synthetic patch results in fragmentation or loss of elastin layers in the area adjacent to the synthetic patch and finally contributes to aneurysm formation in the local region.

The CardioCel patch is well tolerated in low‐pressure areas, but failures happen in high‐pressure areas like the aortic valve, which is attributed to the mechanical mismatch between the elasticity of the native aortic tissue and the patch.^58^, ^124^ A similar phenomenon has been confirmed by Tremblay et al.^166^ In their study, the mismatched mechanical properties between the aorta and prosthetic material were a key factor leading to stenosis. In this case, the stiffer bovine pericardium led to a difference in compliance between two tissues and resulted in intimal proliferation at the suture line.

**FIGURE 5:**
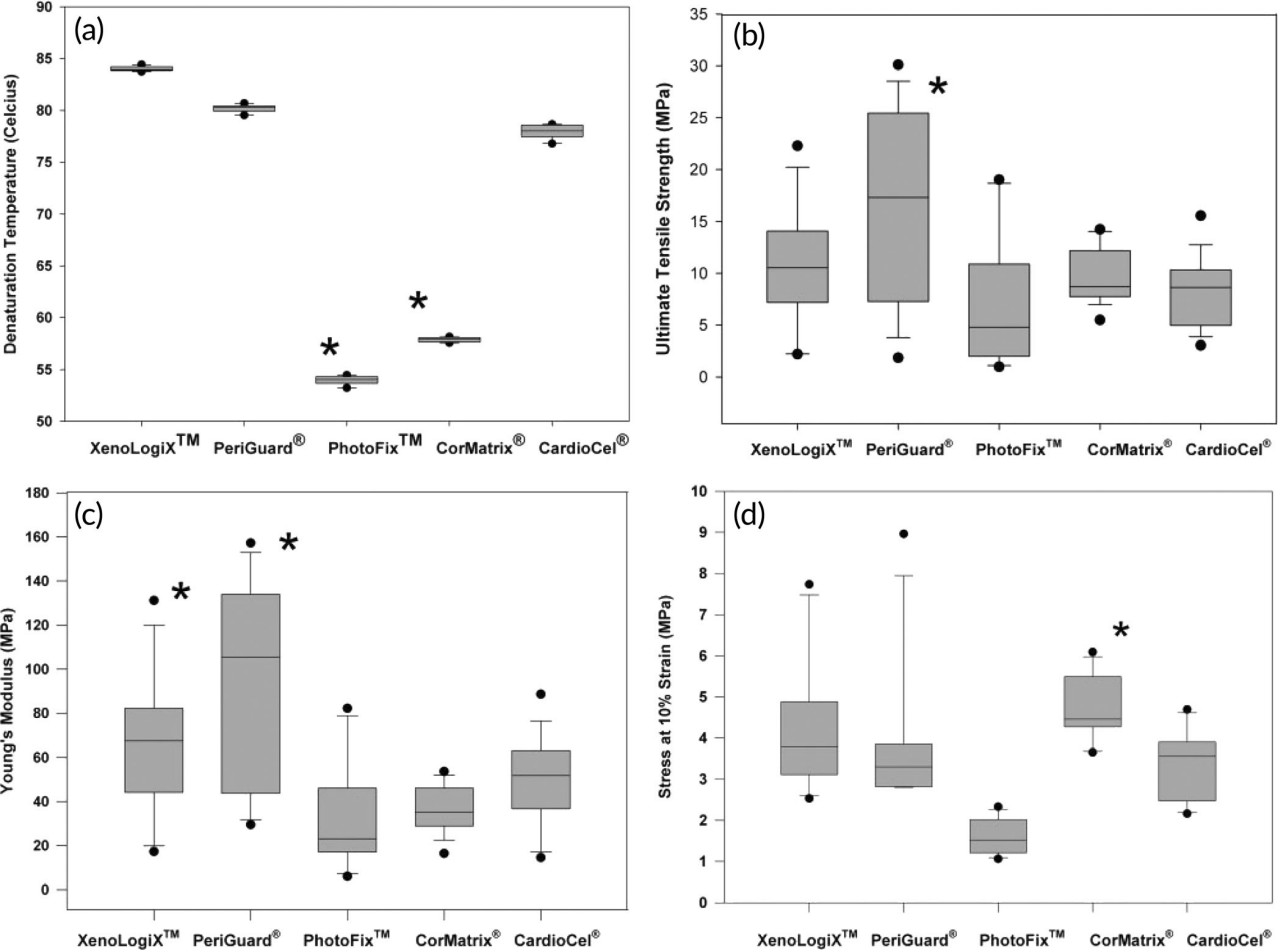
Data are presented as box and whisker plots, with the black lines indicating the medians, the whiskers representing the 10th and 90th percentiles, and asterisk indicating a significant difference (a–d). Reprinted from Reference [167]. Copyright (2018), with permission from Oxford University Press.

A comparison of physical properties in various commonly used patches has been reported by Neethling and colleagues.^167^ This study focused on tensile properties and compared tensile strength, Young's modulus, and stress–strain relationship based on uniaxial measurements. While uniaxial tests provide important qualitative information about native tissue properties, it is better to include biaxial testing or multiple test methods to fully capture the mechanical behavior of planar tissues. Nevertheless, the data from Neethling et al's study are presented as box and whisker plots in Figure 5. The authors believed CardioCel demonstrated optimal physical properties (tensile strength and elasticity), with minimal mineralization potential and superior biocompatibility compared to other patch materials. This comparison of physical properties was conducted in vitro. It may reflect the physical properties of various patches and is used as the reference, but caution is necessary to use this comparison to judge patch performance because (1) no details were disclosed about test conditions and the methods to calculate elasticity (Young's modulus) and flexibility (utilizing stress at a 10% strain) were inadequately defined and lacking the necessary precision and clarity; (2) Applications like aortic leaflet repair require the patch to have similar mechanical performance, like anisotropic behavior and a proper modulus range. Kalejs and his team reported that human aortic valves had a modulus of elasticity as 15.34 ± 3.84 MPa and 1.98 ± 0.15 MPa in the circumferential and radial directions, respectively.^168^ It is noted that the moduli of all tested patches were stiffer than the normal range of the native aortic leaflets (Figure 5c). In addition, the author team also disclosed that this study was supported by Admedus Regen Pty Ltd and the lead author had a conflict of interest as a consultant for Admedus Ltd.

**FIGURE 6:**
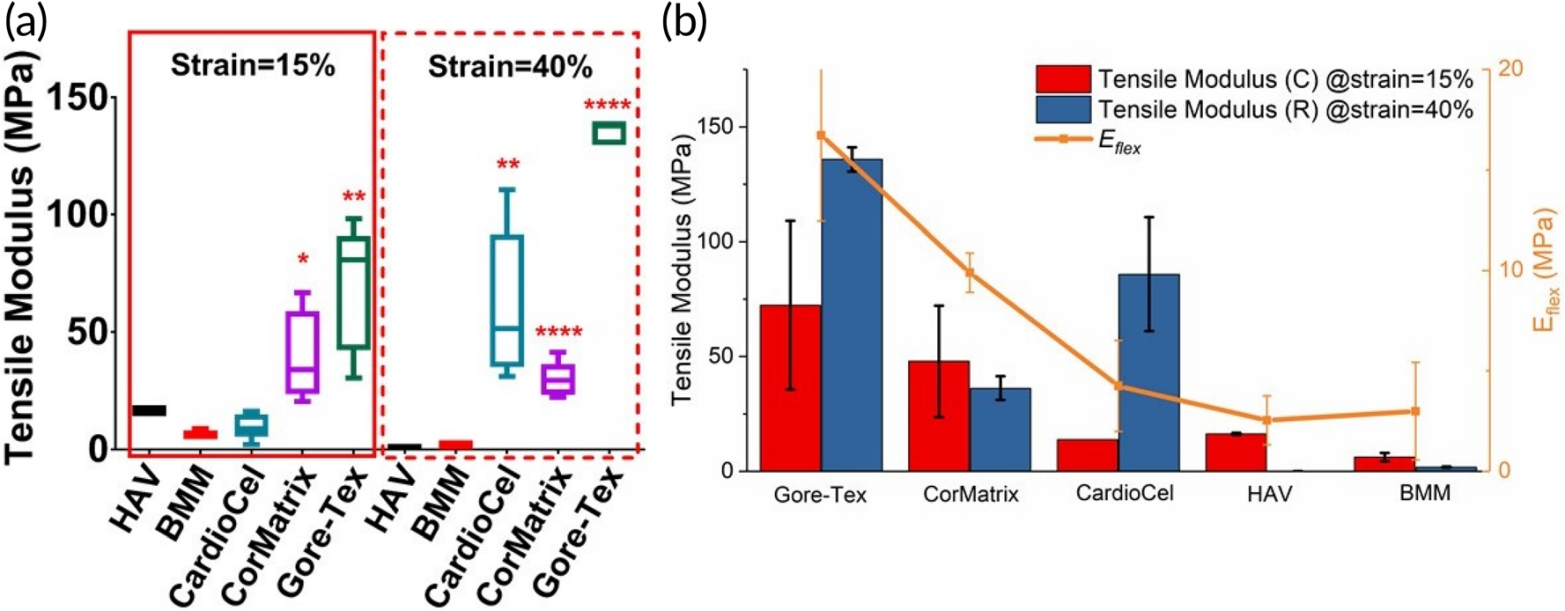
Tensile modulus of all samples (commercial patches, human aortic valve leaflets, referred to as HAV, and the investigated biomimetic multilayered material, referred to as BMM) at strain levels of 15% and 40%. The solid arrow points to the tensile modulus of HAV at 15% strain, indicating the distribution of collagen fibers in the circumferential direction, while the dashed arrow points to the tensile modulus of HAV at 40% strain, exhibiting the existence of a corrugated structure and resulting in lower stiffness in the radial direction. (*p < 0.05, **p < 0.01 and ****p < 0.0001) (a). Tensile modulus (red bars for the circumferential direction and blue bars for the radial direction) and flexural modulus (orange line) of all tested samples (b). Reprinted from Reference [169]. Copyright (2022), with permission from Elsevier.

Sun et al.^169^ conducted a comparison which involves several different patches under the same conditions. In their study, the human aortic valve leaflets have a tensile modulus of 16.34 ± 0.42 MPa in the circumferential direction, similar to Kalejs's study, with a quite low modulus of 0.03 ± 0.01 MPa in the radial direction, indicating the anisotropic nature of native leaflet tissue. Three selected commercial patches, Gore‐Tex, CardioCel and CorMatrix are generally much stiffer (Figure 6a). Gore‐Tex, as a synthetic patch, had the most isotropic and stiffest performance. The two biological commercial products were relatively anisotropic given their biological nature and the presence of residual extracellular matrix fibers, but their anisotropy is still far from that of native leaflet tissue. These three commercial patches also displayed a higher flexural modulus than native tissue (Figure 6b). Nevertheless, the commercial products still possessed much stiffer properties and mismatched mechanical performance, especially in the radial direction. This mechanical discrepancy and resulting remodeling of adjacent tissues, could impact the repair performance and durability of these patches, especially when used in the valvular position.

## 5 SUMMARY

Although current CVPs, from synthetic materials to biological ones, provide generally good outcomes for use in cardiovascular repair or reconstruction, patients, especially children, still face adverse outcomes such as lack of growth, degeneration, calcification, stenosis, inflammatory response, risk of aneurysm formation and recurrent coarctation or stenosis. There is no one‐size‐fits‐all solution for all CVP indications. However, it is encouraging to note that the overall performance of various patches has improved in recent years due to modifications in composition, design, structure, and even the development of new materials, such as TE patches. It may become apparent that a single advanced CVP, while valuable, may not be sufficient to meet the diverse needs of various CHD treatments. Therefore, it is expected that future advancements in CVP technology will result in specialized versions tailored to address specific applications.

## AUTHOR CONTRIBUTIONS

**Mingze Sun:** Writing – original draft; investigation; conceptualization; writing – review and editing; project administration; validation. **V. Reed LaSala:** Conceptualization; investigation; validation; formal analysis; project administration; resources; writing – review and editing. **Caroline Giuglaris:** Methodology; investigation; project administration; writing – original draft. **David Blitzer:** Investigation; validation; supervision; resources. **Sophia Jackman:** Investigation; validation; writing – original draft. **Senay Ustunel:** Validation; project administration; writing – review and editing. **Kavya Rajesh:** Writing – original draft; investigation. **David Kalfa:** Conceptualization; validation; funding acquisition; project administration; writing – review and editing.

## CONFLICT OF INTEREST STATEMENT

The authors declare no conflict of interest.

### PEER REVIEW

The peer review history for this article is available at https://www.webofscience.com/api/gateway/wos/peer-review/10.1002/btm2.10706.

## Data Availability

The data that support the findings of this study are available from the corresponding author upon reasonable request.
